# Not All Acute Abdomen Cases in Early Pregnancy Are Ectopic; Expect the Unexpected: Renal Angiomyolipoma Causing Massive Retroperitoneal Haemorrhage

**DOI:** 10.1155/2016/5643470

**Published:** 2016-06-26

**Authors:** Muhammad Asim Rana, Ahmed F. Mady, Nagesh Jakaraddi, Shahzad A. Mumtaz, Habib Ahmad, Kamal Naser

**Affiliations:** ^1^King's Mill Hospital, Sherwood Forest Hospitals NHS Foundation Trust, Nottinghamshire NG17 4JL, UK; ^2^Department of Intensive Care Medicine, King Saud Medical City, Riyadh 11373, Saudi Arabia

## Abstract

Retroperitoneal haemorrhage (or retroperitoneal haematoma) refers to an accumulation of blood found in the retroperitoneal space. It is a rare clinical entity with variable aetiology including anticoagulation, ruptured aortic aneurysm, acute pancreatitis, malignancy, and bleeding from renal aneurysm. Diagnosis of retroperitoneal bleed is sometimes missed or delayed as presentation is often nonspecific. Multislice CT and arteriography are important for diagnosis. There is no consensus about the best management plan for patients with retroperitoneal haematoma. Stable patients can be managed with fluid resuscitation, correction of coagulopathy if any, and blood transfusion. Endovascular options involving selective intra-arterial embolisation or stent-grafts are clearly getting more and more popularity. Open repair is usually reserved for cases when there is failure of conservative or endovascular measures to control the bleeding or expertise is unavailable and in cases where the patient is unstable. Mortality of patients with retroperitoneal haematoma remains high if appropriate and timely measures are not taken. Haemorrhage from a benign renal tumour is a rarer entity which is described in this case report which emphasizes that physicians should have a wide index of suspicion when dealing with patients presenting with significant groin, flank, abdominal, or back pain, or haemodynamic instability of unclear cause. Our patient presented with features of acute abdomen and, being pregnant, was thought of having a ruptured ectopic pregnancy.

## 1. Case Report

**Figure 1:**
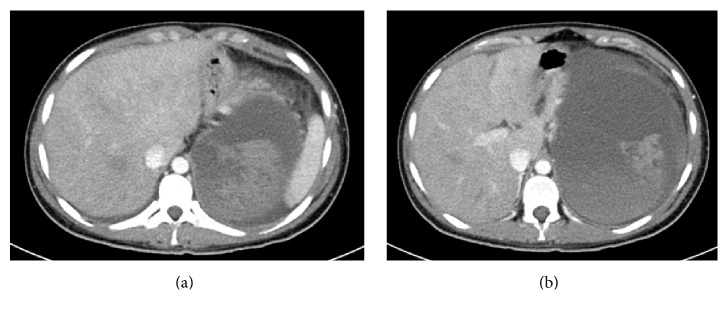
CT abdomen with contrast showing massive retroperitoneal haematoma of different ages.

**Figure 2:**
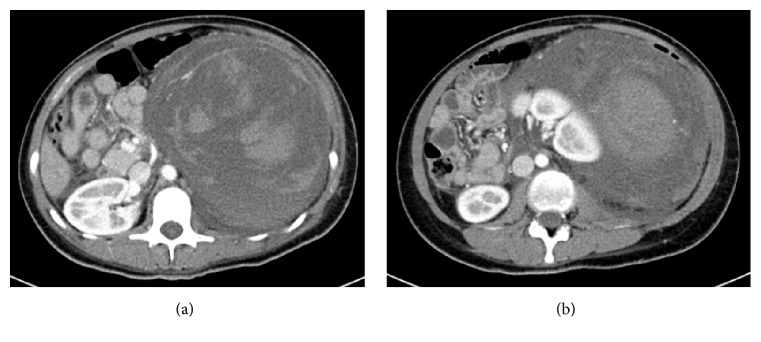
Transverse CT abdomen showing haematoma of varying ages and displaced left kidney.

**Figure 3:**
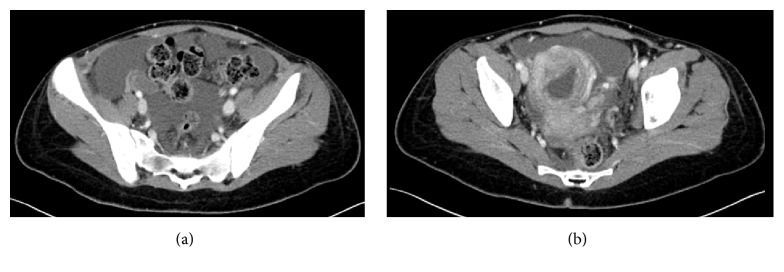
CT pelvis showing haematoma extending to pelvis. Gravid uterus is visible.

A 24-year-old primigravida with 6-week pregnancy was brought into emergency department with severe abdominal pain which started two days earlier and had worsened suddenly in the morning she was admitted. She was referred with a suspected diagnosis of ruptured ectopic pregnancy. Initial examination showed tachycardia of 124 beats per minute, marked pallor, and generalised tenderness in abdomen. The rest of her physical examination was normal. There was no history of vaginal bleeding. Her initial blood counts showed haemoglobin of 4.5 g/L. A FAST (Focused Assessment with Sonography in Trauma) scan appeared to suggest intraperitoneal bleed. Abdominal pain with pallor, low haemoglobin, and positive FAST scan was very suggestive of ectopic pregnancy. Therefore, a CT scan of abdomen was arranged. The scan showed large left retroperitoneal hypodense mass with hyperdense strikes. There was evidence of compression on the left kidney, pancreas, and bowel loops with associated moderate amount of ascites. Findings were consistent with massive retroperitoneal haematoma extending from diaphragm (Figures 1(a) and 1(b)) displacing left kidney (Figures 2(a) and 2(b)) and extending into pelvic cavity. The pregnancy was however found intact within uterus (Figures 3(a) and 3(b)).

**Figure 4:**
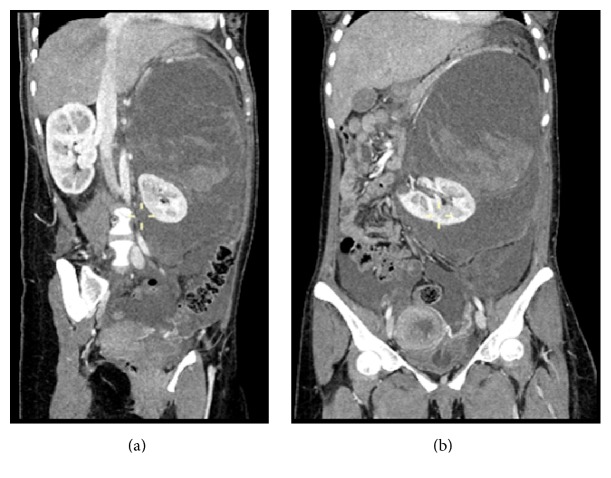
Coronal section showing massive haematoma and displaced left kidney. Cystic lesion in upper pole of left kidney is visible.

**Figure 5:**
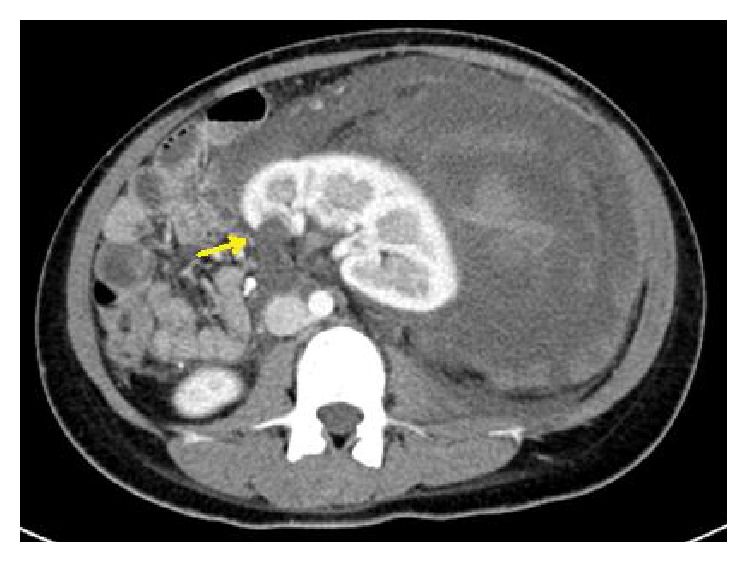
Cystic lesion in left kidney upper pole is clearly visible and marked with yellow arrow.

The haematoma was causing marked displacement of the left kidney and was showing varying shades of hyper- and hypodensities. Varying shades of hyper- and hypodensities were suggestive of haemorrhages of different ages which were periodic and of slow episodes (Figures 4(a) and 4(b)). The main source of bleeding was found to be the apical portion of left kidney which appeared as a cystic lesion in continuity with the haemorrhage (Figure 5).

Patient was kept in intensive care unit under observation and blood was transfused to her to support haemodynamics and achieve a target of Hb% of 10 G/L. Unfortunately, the lesion could not be evaluated with MRI due to technical problem with the MRI scanner.

Initial attempt of embolisation of the lesion failed and next day the haemoglobin level started dropping and therefore a decision was made to intervene. She was taken to operation theatre and partial nephrectomy with evacuation of haematoma was carried out through retroperitoneal approach. Preoperative findings showed a small cystic lesion on upper pole of left kidney which was later confirmed to be renal angiomyolipoma on histopathology. Postoperative period was uneventful and she was discharged back to the care of obstetrician. An uneventful delivery via elective caesarean section was later reported.

This case highlights the silent behaviour of renal angiomyolipomas with slow episodic haemorrhage which can cause damage and displacement of adjacent structures and can actually prove lethal. This patient could have died secondary to haemorrhagic shock. In particular the retroperitoneal haemorrhage could have been easily missed and recurrent haemorrhage on top low haemoglobin could have proven fatal.

## 2. Discussion

Angiomyolipomas of kidneys are the most common benign renal tumours. Majority of them are sporadic (80%) and are typically identified in adults (mean age of presentation: 43 years), with a strong female predilection (F : M ratio of 4 : 1) [1], while remaining 20% are seen in association with the tuberous sclerosis, von Hippel-Lindau syndrome, and neurofibromatosis [2]. In these cases, they are usually identified earlier by the age of 10 years, are larger, and are far more numerous. They are more likely to be fat-poor which may account for their earlier presentation.

Renal angiomyolipomas are often found incidentally when kidneys are scanned for some other reasons but may be present with symptoms like spontaneous retroperitoneal haematoma (like what happened in our case) or other symptoms and signs, for example, palpable abdominal mass, flank pain, recurrent urinary tract infections, haematuria, renal failure, or hypertension due to haemorrhage. The clinical state of shock due to severe haemorrhage from rupture of tumour mass is known as Wunderlich syndrome [2, 3].

Angiomyolipomas are members of the perivascular epithelioid cells tumour group called PEComas and are composed of variable amounts of three components and hence they are named blood vessels (angio), plump spindle cells (myo), and adipose tissue (lipo). Angiomyolipomas are usually benign but they do have the risk of rupture with bleeding. They can also cause secondary damage or destruction of surrounding structures as they grow and pressurise adjacent tissues [3, 4]. A variant of angiomyolipomas called epithelioid angiomyolipoma (as it is composed of epithelial looking cells) has been identified to have some risk of malignant behaviour with some reported cases of metastases [4, 5].

The cornerstone of diagnosis on all modalities is the demonstration of macroscopic fat, however, in the setting of haemorrhage, or when lesions happen to contain little fat, it may be difficult to diagnose with certainty [6].

In ultrasound, these tumours tend to appear as hyperechoic lesions, located in the cortex and with posterior acoustic shadowing [7]. In contrast, enhanced ultrasound tumours have decreased enhancement in centre while they are enhanced more peripherally, a factor which can help in diagnosing the mass [8].

In CT scan, lesions can be seen involving the cortex and can demonstrate macroscopic fat clearly but if lesions are small, differentiation from a small cyst becomes difficult which was the case in our patient [5]. You rarely find calcifications in renal angiomyolipomas.

MRI is an excellent modality to evaluate fat containing lesions and two methods have been described. First is fat saturated technique which demonstrates loss of signals in fat containing areas of lesion and high signal intensities in nonfat containing portions making the differentiation quite easy. Second method employed is in and out of phase imaging which enhances the interface between fat and nonfat components by generating an India ink like artifact [9].

Since angiomyolipomas are hypervascular lesions, DSA (Digital Subtraction Angiography) perhaps can best describe the detailed features like micro- or macroaneurysms, dense arterial network, sharp margins, and absence of arterial-venous shunting of blood.

It is essential to remember that rarely renal cell carcinomas (RCC) have macroscopic fat components and as such the presence of fat is strongly indicative of an angiomyolipoma, but not pathognomonic. Since macroscopic fat in RCC almost always occurs in the presence of ossification/calcification, the absence of ossification/calcification on imaging is in favour of angiomyolipoma.

Angiomyolipomas if small usually require no therapy although follow-up is recommended to assess their growth.

Larger angiomyolipomas or those that have been symptomatic can be electively embolised and/or resected with a partial nephrectomy. Lesions with retroperitoneal haemorrhage often require emergency embolisation as a life saving measure [10]. In our case, the patient underwent surgery after failed attempt of embolisation and partial nephrectomy was done.
